# Mortality and causes of death in a population with blindness in Korea: A longitudinal follow-up study using a national sample cohort

**DOI:** 10.1038/s41598-020-61805-6

**Published:** 2020-03-17

**Authors:** Hyo Geun Choi, Min Joung Lee, Sang-Mok Lee

**Affiliations:** 1 Department of Otorhinolaryngology-Head & Neck Surgery, Hallym University College of Medicine, Anyang, Republic of Korea; 2 Department of Ophthalmology, Hallym University College of Medicine, Anyang, Republic of Korea; 3 Department of Ophthalmology, Catholic Kwandong University College of Medicine, Gangneung-si, Gangwon-do 25601 Republic of Korea; 4 Department of Cornea, External Disease & Refractive Surgery, HanGil Eye Hospital, Incheon, 21388 Republic of Korea

**Keywords:** Vision disorders, Risk factors

## Abstract

The influence of visual impairment and blindness on the risk of mortality has been reported in diverse cohort studies. However, the results reported have varied from nonsignificant to significant associations. In the present study, we evaluated the influence of blindness on the risk of mortality from 2002 to 2013 using a longitudinal database with a national sample cohort provided by the Korean National Health Insurance Service. Of a total of 1,125,691 subjects, 1,279 subjects who were registered as blind were enrolled, and 5,116 control participants were matched at a 1:4 ratio for age, sex, income, region of residence, and medical histories of hypertension, diabetes mellitus and dyslipidemia. The life/death information contained in this dataset was used for the analysis; this information was originally recorded by the medical doctors on the death certificates of the participants. The percentage of total deaths during the mean follow-up period of 111.0 ± 41.6 months was 28.1% in the blindness group and 19.7% in the matched control group. The risk of mortality was significantly higher in the blindness group than in the control group according to the Cox proportional hazards model with additional adjustments for ischemic heart disease, stroke, and depression (adjusted hazard ratio [HR] of mortality = 1.54, 95% confidence interval [CI] = 1.37–1.74, P < 0.001). In the subgroup analyses, the adjusted HRs for mortality were significantly higher in the blindness group than in the control group regardless of age (young defined as <60 years old vs old defined as ≥60 years old) and sex. The percentage of death due to metabolic diseases and genitourinary diseases was higher in the blindness group than in the matched control group.

## Introduction

Sensory impairments are common among the elderly population^1–3^. In particular, visual impairment, including blindness, negatively impacts functional independence, cognition, and mental health, consequently resulting in reduced quality of life^3–7^. According to the criteria of the World Health Organization (WHO), visual impairment is defined as presenting vision worse than 20/60 (or 6/18) in the better seeing eye and blindness as vision worse than 20/400 (or 3/60) in the better seeing eye^1,8^. According to population-based studies, the reported prevalence of visual impairment ranges from 0.35% to 2.54%, and that of blindness ranges from 0.1% to 1.02% according to the WHO criteria^9^. The influence of visual impairment and blindness on the risk of mortality has been reported in diverse cohort studies, but the results have varied from nonsignificant to significant associations, and the relative risk (RR) has varied from 1.17 to 3.60^8,10–19^. The risk of mortality associated with visual impairment was well summarized in a recent meta-analysis including 29 studies with a total of 269,839 participants and 67,061 deaths^8^. Of the 29 included studies, 17 studies reported a significant association between visual impairment and the risk of mortality, but the rest of the studies failed to identify a significant relationship^8^. Pooling the results was challenging because the criteria for visual impairment differed among the studies, and thus, the authors obtained a conclusion by comparing the no visual impairment group (control) with the highest visual impairment level group reported in each study^8^. The pooled RR in their report was 1.36 (95% CI = 1.25–1.46) with this method^8^. The association between blindness and the risk of mortality can be regarded as an extension of visual impairment because blindness is the most severe form of visual impairment. However, some reports have shown that the risk of mortality was higher in only the visual impairment group (moderate to severe low vision) and not in the blindness (profound vision loss) group^15,20,21^.

The purpose of this study is to evaluate the association between the risk of mortality and blindness, as defined by the WHO criteria, and to compare the causes of death between the blindness population and their matched controls using a representative sample cohort dataset provided by the Korean National Health Insurance Service (KNHIS), which includes data on individual disabilities, life/death information, and causes of death (for dead individuals) and is synchronized with governmental data.

## Results

**Figure 1:**
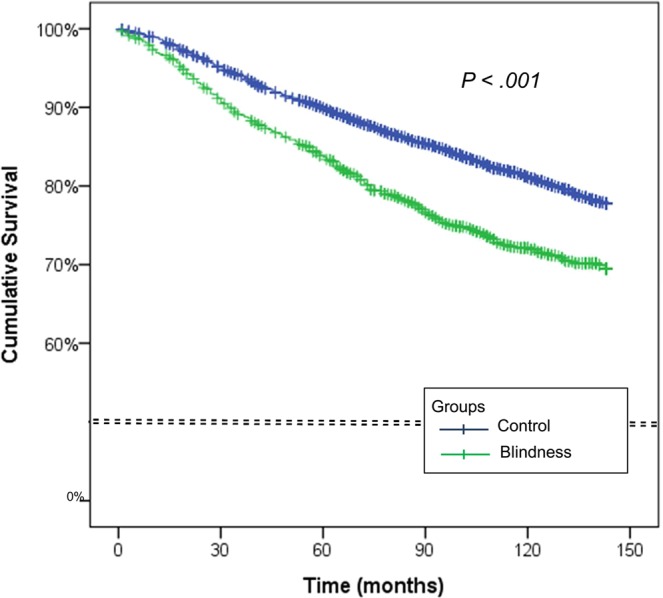
Kaplan–Meier survival curves for the blindness and control groups. The blindness group (green line) had a lower cumulative survival rate than the control group (blue line) (*P* < 0.001).

The detailed matched data and the distribution of the participants according to age group, sex, income level group, region of residence category, hypertension, diabetes, and hyperlipidemia are shown in Table 1. The percentage of total deaths during a mean follow-up of 111.0 ± 41.6 months was 28.1% (360 deaths out of 1,279 persons) in the blindness group and 19.7% (1,008 deaths out of 5,116 persons) in the matched control group (Table 1). After adjustment for age group, sex, income group, region of residence category, treatment history of comorbidities including hypertension, diabetes, hyperlipidemia, ischemic heart disease, stroke, and history of diagnosis of depression by a psychiatrist using a Cox proportional hazard model, the crude hazard ratio (HR) for mortality was 1.51 (95% CI = 1.34–1.71, *P* < 0.001) and the adjusted HR for mortality was 1.54 in the blindness group (95% CI = 1.37–1.74, *P* < 0.001) compared with the matched control group (Table 2). In the Kaplan-Meier survival analysis, the blindness group showed a lower cumulative survival rate than the control group (log-rank test, *P* < 0.001, Fig. 1).

**Table 1:** General Characteristics of Participants.

Characteristics	Blindness vs control (matched 1:4)		
	Blindness (n, %)	Control group (n, %)	P-value
Age (years old)			1.000
0–4	15 (1.2)	60 (1.2)	
5–9	9 (0.7)	36 (0.7)	
10–14	15 (1.2)	60 (1.2)	
15–19	19 (1.5)	76 (1.5)	
20–24	22 (1.7)	88 (1.7)	
25–29	28 (2.2)	112 (2.2)	
30–34	49 (3.8)	196 (3.8)	
35–39	53 (4.1)	212 (4.1)	
40–44	72 (5.6)	288 (5.6)	
45–49	116 (9.1)	464 (9.1)	
50–54	123 (9.6)	492 (9.6)	
55–59	128 (10.0)	512 (10.0)	
60–64	144 (11.3)	576 (11.3)	
65–69	152 (11.9)	608 (11.9)	
70–74	129 (10.1)	516 (10.1)	
75–79	96 (7.5)	384 (7.5)	
80–84	56 (4.4)	224 (4.4)	
85+	53 (4.1)	212 (4.1)	
Sex			1.000
Male	637 (49.8)	2,548 (49.8)	
Female	642 (50.2)	2,568 (50.2)	
Income			1.000
1 (lowest)	350 (27.4)	1,400 (27.4)	
2	109 (8.5)	436 (8.5)	
3	64 (5.0)	256 (5.0)	
4	90 (7.0)	360 (7.0)	
5	83 (6.5)	332 (6.5)	
6	68 (5.3)	272 (5.3)	
7	89 (7.0)	356 (7.0)	
8	89 (7.0)	356 (7.0)	
9	89 (7.0)	356 (7.0)	
10	118 (9.2)	472 (9.2)	
11 (highest)	130 (10.2)	520 (10.2)	
Region of residence			1.000
Urban	564 (44.1)	2,256 (44.1)	
Rural	715 (55.9)	2,860 (55.9)	
Hypertension	645 (50.4)	2,580 (50.4)	1.000
Diabetes	431 (33.7)	1,724 (33.7)	1.000
Hyperlipidemia	276 (21.6)	1,104 (21.6)	1.000
Ischemic heart disease	103 (8.1)	412 (8.1)	1.000
Stroke	228 (17.8)	810 (15.8)	0.084
Depression	228 (10.4)	810 (8.7)	0.055
Death	360 (28.1)	1,008 (19.7)	<0.001^*^

^*^Chi-square test. Significance at P < 0.05.

**Table 2:** Crude and adjusted hazard ratios (95% confidence interval) of blindness for mortality.

Characteristics	Crude	P-value	Adjusted^†^	P-value
Blindness		<0.001^*^		<0.001^*^
Yes	1.51 (1.34–1.71)		1.54 (1.37–1.74)	
No	1.00		1.00	

^*^Cox proportional hazard regression model, significance at P < 0.05.

^†^Adjusted model for age, sex, income, region of residence, hypertension, diabetes, hyperlipidemia, ischemic heart disease, stroke, and depression histories.

The causes of death are presented in Table 3. The percentage of death due to metabolic diseases and genitourinary diseases were significantly higher in the blindness group than in the matched control group (metabolic disease: odds ratio [OR] = 3.59, 95% CI = 2.55–5.04, *P* < 0.001; genitourinary disease: OR = 6.10, 95% CI = 3.23–11.51, *P* < 0.001; Table 3).

**Table 3:** The difference in the percentage of death between the blindness and control groups according to cause of death.

Cause of death	Blindness vs Control (matched 1:4)			
	Blindness group (n = 1,279)	Control group (n = 5,116)	Odd ratio (95% CI)	P-value
All of death (n, %)	360 (28.1)	1,008 (19.7)	1.60 (1.39–1.84)	<0.001^*^
Infection (n, %)	13 (1.0)	25 (0.5)	2.09 (1.07–4.10)	0.028
Neoplasm (n, %)	49 (3.8)	250 (4.9)	0.78 (0.57–1.06)	0.110
Metabolic disease (n, %)	64 (5.0)	74 (1.4)	3.59 (2.55–5.04)	<0.001^*^
Mental diseases (n, %)	8 (0.6)	23 (0.4)	1.39 (0.62–3.12)	0.418
Neurologic disease (n, %)	6 (0.5)	25 (0.5)	0.96 (0.39–2.35)	0.928
Circulatory disease (n, %)	79 (6.2)	241 (4.7)	1.33 (10.3–1.73)	0.031
Respiratory disease (n, %)	24 (1.9)	87 (1.7)	1.11 (0.70–1.74)	0.667
Digestive disease (n, %)	13 (1.0)	46 (0.9)	1.13 (0.61–2.10)	0.695
Genitourinary disease (n, %)	24 (1.9)	16 (0.3)	6.10 (3.23–11.51)	<0.001^*^
Trauma (n, %)	14 (1.1)	65 (1.3)	0.87 (0.48–1.55)	0.626
Others^†^ (n, %)	4 (0.3)	16 (0.3)	1.00 (0.33–3.00)	0.777
NEC (n, %)	62 (4.8)	140 (2.7)	1.81 (1.33–2.46)	<0.001^*^

^*^Chi-square test or Fisher’s exact test. Significance at false discovery rate adjusted P < 0.05.

^†^Others includes Blood and Immune diseases, Skin diseases, Muscular diseases, and Congenital anomaly related.

CI: confidence interval, NEC: Not elsewhere classified.

In the subgroup analyses according to age and sex, the adjusted HRs for mortality were higher for the blind group than for the matched control group in all four subgroups (Table 4). For the young subgroup (age <60 years old), the adjusted HR was 2.23 (95% CI = 1.72–2.89, *P* < 0.001) and for the old subgroup (age ≥60 years old), the adjusted HR was 1.41 (95% CI = 1.23–1.61, *P* < 0.001). When the old age group was subdivided into three subgroups (60~69 years old, 70~79 years old, and ≥80 years old), the adjusted HRs were 1.95 (95% CI = 1.53–2.49, n = 1,480, *P* < 0.001), 1.29 (95% CI = 1.04–1.61, n = 1,125, *P* = 0.022), and 1.11 (95% CI = 0.86–1.43, n = 545, *P* = 0.447) respectively (Supplemental Table 1). All the age groups except the oldest age subgroup (≥80 years old) had significantly increased adjusted HRs, which were progressively less elevated with increasing age. For the male subgroup, the adjusted HR was 1.43 (95% CI = 1.20–1.69, *P* < 0.001) and for the female subgroup, the adjusted HR was 1.70 (95% CI = 1.43–2.02, *P* < 0.001).

**Table 4:** Subgroup analyses of adjusted hazard ratios (95% confidence interval) of blindness for mortality according to age (young: <60 years old; old: ≥60 years old) and sex.

Characteristics	Crude	P-value	Adjusted^†^	P-value
Young (<60 years old)	**N** = **3,245**			
Blindness		<0.001^*^		<0.001^*^
Yes	2.23 (1.72–2.88)		2.23 (1.72–2.89)	
No	1.00		1.00	
Old (≥60 years old)	**N** = **3,150**			
Blindness		<0.001^*^		<0.001^*^
Yes	1.41 (1.23–1.61)		1.41 (1.23–1.61)	
No	1.00		1.00	
Male	**N** = **3,185**			
Blindness		<0.001^*^		<0.001^*^
Yes	1.41 (1.19–1.67)		1.43 (1.20–1.69)	
No	1.00		1.00	
Female	**N** = **3,210**			
Blindness		<0.001^*^		<0.001^*^
Yes	1.64 (1.38–1.94)		1.70 (1.43–2.02)	
No	1.00		1.00	

^*^Cox proportional hazard regression model, significance at P < 0.05.

^†^Adjusted model for age, sex, income, region of residence, hypertension, diabetes, hyperlipidemia, ischemic heart disease, stroke, and depression histories.

## Discussion

In the present study, we evaluated the risk of mortality in a population with registered visual disability grades I–II (i.e. profound visual impairment), which is compatible with the criteria for blindness by the WHO, in a nationwide cohort. The results showed that blindness significantly increased the risk of mortality, with an adjusted HR of 1.54 (95% CI = 1.37–1.74) compared with the control group, with a mean follow-up of 111.0 months after adjusting for age, sex, income, region of residence, and past medical histories (hypertension, diabetes, dyslipidemia, ischemic heart disease, stroke, and depression). Although the hazard of death was significantly increased by blindness regardless of age or sex, a tendency for an even greater hazard was observed in the young subgroup (adjusted HR = 2.23 for young; adjusted HR = 1.41 for old; all *P* < 0.001, Table 4). The relatively high adjusted HR for the young subgroup may be caused by the extraordinarily low percentage of death (6.4%) in the control population of this subgroup (Supplemental Table 2). The hazard of death in the blindness group tended to decrease with age when the old subgroup was subdivided by 10-year intervals. Similar trends were observed in the pooled risk for the group with visual impairment through a meta-analysis and in a retrospective matched cohort study targeting the working age blind population (aged 18–65 years)^8,22^.

The finding of an increased HR of mortality in the blindness group is quite similar to the result from a previous cohort study from India (an adjusted HR = 1.5, 95% CI = 1.1–2.0, blindness criteria: presenting visual acuity <6/60 or a central visual field less than 20° in the better seeing eye) and a population-based study using social security files from a region in Germany (standardised mortality ratio = 1.41, 95% CI = 1.28–1.54, blindness criteria: binocular best corrected visual acuity ≤1/50 or a central visual field less than 5°), though the criteria were different from those in this study^18,23^. A higher OR was reported for the blindness group in rural Africa (OR = 3.33, 95% CI = 1.03–11.04, blindness criteria: 6/60 or less in the better seeing eye), where vision may be more important for survival^24^. In contrast, there are some studies that could not find any association between mortality and blindness; these studies were conducted in England, Singapore, Australia, Iceland, and Kenya^15,20,21,25,26^. Especially in studies from England, Singapore, and Kenya, mortality was not significantly higher in the blindness group, although the risk of mortality was significantly higher in the visual impairment group than in the control group in the same studies^15,20,21^. Increased social contact experienced by blind individuals, considering that a blind person is more likely to live with a sighted friend or relative than is a less seriously visually impaired person, was suggested as a possible explanation for these results^20^. Because previous studies showed such contradictory results, the authors of this study planned to elucidate the association between blindness and mortality using a national sample cohort comprised of one million subjects, which were selected to represent the entire South Korean population (50 million) using randomized stratified systematic sampling methods^27^. In this study, the risk of mortality was significantly higher by 1.54 times in the blindness group compared with the control group in the Korean population.

Regarding the causes of death, metabolic diseases and genitourinary diseases had higher ORs in the blindness group than in the matched control group. In the analysis of the subcategories, 98.4% of the deaths due to metabolic diseases (63 out of 64 deaths) were related to diabetes, and all deaths due to genitourinary diseases (all 24 deaths) were related to renal failure in the blindness group. Considering previous reports of an increased prevalence of diabetes in visually impaired individuals in Korea (adjusted OR = 2.273, 95% CI = 1.503–3.437) and reports on the relationship between mortality and diabetic retinopathy, increased mortality in the blindness group may be mainly associated with diabetes^9,16,19,21,28–31^. In a retrospective cohort analysis of indigenous Australians, of the diverse disease-specific causes of visual impairment, only visual impairment due to diabetic retinopathy increased the 10-year mortality after adjustments for age, sex and the presence of systemic hypertension (HR = 1.70, 95% CI = 1.00–2.87, *P* = 0.049)^19,31^. Although the presence of diabetes was adjusted for by matching in this study, the severity of diabetes could not be addressed. The significantly increased ORs of metabolic diseases in the blindness group implies that the severity of diabetes may be related to the relatively higher mortality in this group. The type of diabetes was not analyzed separately considering the low prevalence of type I diabetes (0.041~0.047%) in Korea^32,33^.

However, the increased mortality due to genitourinary diseases is difficult to interpret considering that renal failure caused by diabetic nephropathy should be classified as a metabolic disease. Age-related macular degeneration, which is also an important cause of blindness in the elderly population, may explain this result because it has been reported to have a positive relationship with chronic kidney diseases^34,35^. Primary mitochondrial disorders are also known to cause both blindness and renal problems^36^. Conversely, patients undergoing chronic hemodialysis with advanced kidney diseases are known to suffer from vision-affecting eye problems such as retinal hemorrhage, retinal detachment, glaucoma, cataract, and optic neuropathy related to increased blood pressure, uremic state, combined anemia, intradialytic hypotension, heparinization during dialysis, and dialysis itself^37^. Although further detailed analysis is difficult in this dataset, increased percentage of death from renal failure other than diabetic nephropathy in the blind population could have been caused by a combination of these correlations between kidney diseases and eye diseases. Further study is needed to assess the relationship between blindness and mortality from renal failure.

Interestingly, trauma and depression, which may be suspected as possible causes of death in blind people, have not been identified as significantly more common causes of death compared to controls. Taken together, these analysis results suggest that in Korea, the increased risk of mortality in the blindness population is caused by comorbidities, mainly diabetes and kidney disease, rather than blindness itself.

This study has several strengths and limitations. The greatest strengths of this study were that it was a large-scale study consisting of one million subjects, and the dataset was a nationally representative sample cohort provided by the nationwide compulsory national health insurance system^2,27^. To identify the exact risk of mortality, minimizing missing information regarding death is important; thus, an accurate analysis is possible if the dataset includes compulsory death information collected by the government to analyze demographics. The linkage of the information on death to the dataset used in this study was advantageous because the information was from legally enforced notices. Causes of death and dates are recorded by medical doctors on the death certificates. Additionally, the dataset was useful because it did not allow missing data; all Korean citizens are identified by a 13-digit resident registration number from birth to death and are required to enroll in the KNHIS^7,38^. Another advantage of this study was the qualified evaluation of visual function. Because the registration of disability is related to governmental benefits, the government tightly controls the evaluation system for visual function, including best corrected visual acuity, visual field tests, manifest refraction, anterior segment photography, fundus photography, visual evoked potential, electroretinography, and optical coherence tomography, if needed^2^. After the submission of the required data, the processes of reviewing and confirming the data were performed by ophthalmologists hired by the government^2,7^. The other advantage of this study was that mortality was analyzed in all age groups; many previous cohort studies enrolled only adult populations^3,15,31^. The HRs of mortality were significantly higher in both the young and the old age subgroups of the blind cohort in this study. The fourth advantage of this study is the case-control design, which strongly controlled for confounding factors such as age, sex, socioeconomic condition, and diabetes. When the controls were selected by matching only 4 factors (age group, sex, income group, and region of residence group), the HR for mortality was 4.36 (95% CI = 3.74–5.08, data not shown), even after adjustments for hypertension, diabetes, hyperlipidemia, ischemic heart disease, and stroke histories using a Cox proportional hazard regression model in the blindness group. However, when the controls were selected by matching 7 factors (the previous 4 factors + medical histories of hypertension, diabetes, and hyperlipidemia), the adjusted HR for mortality decreased to 1.54 (95% CI = 1.37–1.74). Changes in the adjusted HR were more pronounced in less severe visual impairment group (adjusted HR = 5.33, 95% CI = 4.71–6.03 by matching 4 factors vs adjusted HR = 1.01, 95% CI = 0.92–1.10 by matching 7 factors, data not shown). The marked change in the HR for mortality due to the additional factors used for matching suggested the limitation of the statistical control of confounding factors and therefore indicated that controlling for the highly suspected variables by matching rather than by statistical adjustment was a possible advantage of this study. However, ischemic heart disease, stroke, and depression were adjusted by statistical adjustment in the Cox proportional hazard model, rather than by matching, to avoid selection bias. Because these three diseases are found in less than 20% of the total population in this cohort, sufficient participants with these conditions may not be available to serve as matched controls, leading to the exclusion of individuals from the study and, therefore, selection bias.

There were also several limitations. Due to the case-control comparison design of the study, the exact prevalence and incidence could not be calculated in this study. For this reason, we used the term “percentage of death” instead of “mortality rate”. Second, the presenting visual acuity or the presence of neovascular age-related macular degeneration, which are reported to have relatively superior predictive value for mortality, could not be used in this study because of limitations in the disability registration data regarding visual function^15,21,39^. Additionally, individuals with treatable transient blindness conditions, especially those caused by severe cataracts, could not be included in the blindness group in this study, in contrast with previous papers^15,18,31,40^. However, considering the easy accessibility of cataract surgery in South Korea, this limitation was not a serious disadvantage of this study. The exact average age could not be calculated because the age data were provided as categorized values in five-year intervals. However, the age distribution of participants is shown as the number of participants in each age group in Table 1.

In summary, this study demonstrated that the risk of mortality was significantly higher (1.54 times) in the blindness group compared with the control group in the Korean population. Regarding causes of death, deaths from metabolic diseases and genitourinary diseases, represented by diabetes and renal failure, were significantly more common in the blindness group than in the control group.

## Methods

### Study population and data collection

The Institutional Review Board (IRB)/Ethics Committee of Hallym University (2017-I102) approved the use of these data. The need for written informed consent was waived by the IRB.

This national cohort study relied on data from the Korean Health Insurance Review and Assessment Service - National Sample Cohort (HIRA-NSC)^7,27^. A detailed description of the dataset was provided in our previous study^7^. In Korea, it is a legal requirement for a notice of death to be provided to an administrative entity before a funeral can be held. The causes and dates of death are recorded by medical doctors on death certificates.

### Participant selection

Out of 1,125,691 cases with 114,369,638 medical claim codes, we included participants who were registered as benefit recipients due to visual disability in the Ministry of Health and Welfare. In Korea, patients must submit a medical certificate issued by an ophthalmologist regarding their best corrected visual acuity, visual field, and possible reason for visual impairment before being registered as a benefit recipient. With properly documented evidence of visual impairment, an assessment committee discusses the feasibility of the registration and the appropriate grade of the benefit according to the degree of visual impairment of the applicant. Though the benefits in this system are typically divided into 6 grades (I–VI) according to the degree of impairment, the data provided in this dataset are categorized into profound visual impairment (grades I–II) vs moderate to severe visual impairment (grades III–VI). The profound visual impairment presented in this dataset required a best corrected visual acuity <20/400 in the better eye, which is compatible with the “blindness” definition of the WHO^1,2,8^. Thus, 1,292 participants who met the criteria for blindness were selected from this dataset.

**Figure 2:**
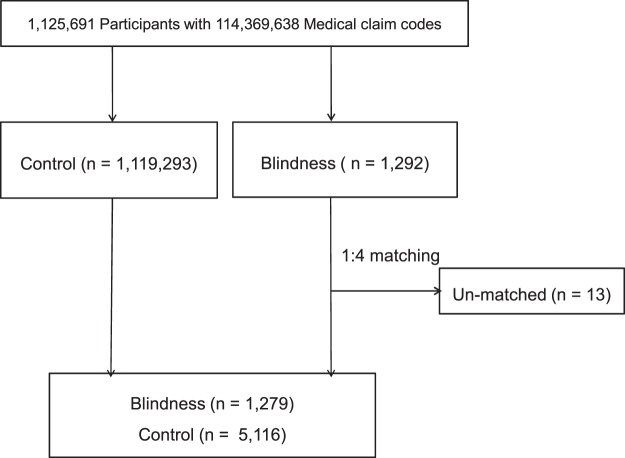
A flowchart of the participant selection process that was used in the present study. Out of a total of 1,125,691 participants, 1,292 participants with blindness were selected. The participants with blindness were matched at a 1:4 ratio with a control group that did not present visual impairment. Unmatched participants with blindness were excluded (n = 13). Finally, 1,279 participants with blindness and 5,116 control participants were included.

Participants with blindness were matched with participants (control group) who had never been registered for any disabilities, including visual disabilities, from 2002 through 2013 at a 1:4 ratio. The participants were matched for age group, sex, income group, region of residence group, and medical histories of hypertension/diabetes mellitus/dyslipidemia. Participants in the control group were sorted using a random number order and then selected from top to bottom to prevent selection bias, as described in our previous paper^7^. The matched control participants were assumed to have been enrolled in the study at the same time as each matched blind participant. Therefore, participants in the control group who died prior to matching with the blind participants were excluded. Accordingly, blind participants for whom we were not able to identify a sufficient number of matching participants were excluded (n = 13). Finally, 1:4 matching resulted in the inclusion of 1,279 participants with blindness and 5,116 control participants (Fig. 2). However, they were not matched for past medical histories of ischemic heart diseases, stroke, and depression, which were present in less than 20% of the total population in this cohort, because strict matching increased the loss of blindness participants due to a lack of sufficient matched control participants.

### Variables used for matching

The variable groupings were formed as described in our previous articles^7,38^. In brief, the age groups were categorized into 18 groups with 5-year intervals, and the income groups were categorized into 11 classes (from class 1 [lowest income] to class 11 [highest income]). The region of residence was grouped into urban and rural categories.

The causes of death were categorized following the Korean standard classification of diseases (KCD), which was developed based on the International Statistical Classification of Diseases and Related Health Problems (ICD) by the WHO^41,42^. Though 14 categories had reported deaths, we merged three categories with limited numbers of deaths (Diseases of the blood and blood-forming organs and certain disorders involving the immune mechanism, Diseases of the skin and subcutaneous tissue, and Diseases of the musculoskeletal system and connective tissue) into one, and finally, the causes of death were categorized into 12 classifications (Supplemental Table 3, Table 3).

The past medical histories of the participants were evaluated using ICD-10 codes. To ensure the accuracy of the diagnosis, hypertension (I10 and I15), diabetes (E10-E14), and hyperlipidemia (E78) were considered positive if the participants were treated ≥2 times. Ischemic heart disease (I24 and I25) and stroke (I60-I66) were considered positive if the participants were treated ≥1 time. Depression was defined using the ICD-10 codes F31 (bipolar affective disorder) through F39 (unspecified mood disorder) recorded by a psychiatrist.

### Statistical analyses

Chi-square tests were used to compare the general characteristics between the blindness and control groups. A chi-square test or Fisher’s exact test was used to compare the percentage of death between the blindness group and the control group according to the categories of cause of death. In this analysis, to adjust for the expected value of the incorrectly declined null hypothesis, the false discovery rate was used.

To analyze the cumulative survival rate of the blindness and control groups, Kaplan-Meier survival analysis with the log-rank test was used. To analyze the HR for mortality in the blindness population compared with the control population, a Cox proportional hazard model was used. In this analysis, crude (simple) and adjusted (age, sex, income, region of residence, hypertension, diabetes, hyperlipidemia, ischemic heart disease, stroke and depression histories) models were used. In the subgroup analyses, we divided the participants according age (young: <60 years old; old: ≥60 years old) and sex. Then, the old age group was subdivided into three subgroups (60~69 years old, 70~79 years old, and ≥80 years old) to check the trend in the adjusted HRs according to age in the elderly population.

Two-tailed analyses were conducted, and *P*-values less than 0.05 were considered to indicate significance. The results were statistically analyzed using SPSS v. 21.0 (IBM, Armonk, NY, USA).

## Supplementary information

Supplemental Tables 1-3.

## Data Availability

The unprocessed raw data are available at nhiss.nhis.co.kr. The datasets generated during and/or analysed during the current study are not publicly available because the authors do not have permission to share the data but are available from the corresponding author on reasonable request.
