# Supplementary material for: Mortality and causes of death in a population with blindness in Korea: A longitudinal follow-up study using a national sample cohort
Source: Sci Rep. 2020 Mar 17;10:4891. doi: 10.1038/s41598-020-61805-6 (PMC7078281; doi:10.1038/s41598-020-61805-6)
Supplement: Supplementary file 1 — Supplemental Tables 1-3. [file 41598_2020_61805_MOESM1_ESM.pdf]

Mortality and causes of death in a population with blindness in Korea: A longitudinal follow-up study using a national sample cohort

Hyo Geun Choi, MD[1], Min Joung Lee, MD[2], Sang Mok Lee, MD[3,4]

[1]*Department of Otorhinolaryngology-Head & Neck Surgery, Hallym University College of Medicine, Anyang, Republic of Korea*

[2]*Department of Ophthalmology, Hallym University College of Medicine, Anyang, Republic of Korea*

[3]*Department of Ophthalmology, Catholic Kwandong University College of Medicine, Gangneung-si, Gangwon-do 25601, Republic of Korea*

[4]*Department of Cornea, External Disease & Refractive Surgery, HanGil Eye Hospital, Incheon 21388, Republic of Korea*

Supplemental Table 1. Detailed analyses of adjusted hazard ratios (95% confidence interval) of blindness for mortality according to age in the old age subgroup (≥ 60 years old)

Characteristics	Crude	P-value	Adjusted†	P-value
**60~69 years old**		**N = 1,480**		
Blindness		< 0.001*		< 0.001*
Yes	1.97 (1.55-2.52)		1.95 (1.53-2.49)	
No	1.00		1.00	
**70~79 years old**		**N = 1,125**		
Blindness		0.035*		0.022*
Yes	1.26 (1.02-1.57)		1.29 (1.04-1.61)	
No	1.00		1.00	
**≥ 80 years old**		**N = 545**		
Blindness		0.447		0.484
Yes	1.11 (0.86-1.43)		1.10 (0.85-1.43)	
No	1.00		1.00	

* Cox-proportional hazard regression model, Significance at P < 0.05

† Adjusted model for age, sex, income, region of residence, hypertension, diabetes, hyperlipidemia, ischemic heart disease, stroke, and depression histories

Supplemental Table 2. Subgroup analyses of the percentage of death and odd ratios according to age (young: < 60 years old; old: ≥ 60 years old) and sex

Subgroup	Total number (n)	Number of death (%)		Odd ratio (95% CI)	P-value
**Young** (< 60 years old)			**N = 3,245**		
Blindness	649	88 (13.6)		2.31	< 0.001*
Control	2,596	165 (6.4)		(1.76-3.04)	
**Old** (≥ 60 years old)			**N = 3.150**		
Blindness	630	272 (43.2)		1.51	< 0.001*
Control	2,520	843 (33.5)		(1.26-1.81)	
**Male**			**N = 3,185**		
Blindness	637	179 (28.1)		1.46	< 0.001*
Control	2,548	538 (21.1)		(1.20-1.78)	
**Female**			**N = 3,210**		
Blindness	642	181 (28.2)		1.75	< 0.001*
Control	2,568	470 (18.3)		(1.44-2.14)	

* Chi-square test. Significance at false discovery rate adjusted P < 0.05

CI: confidence interval

Supplemental Table 3. Cause of death classified following Korean Standard Classification of Diseases.

Category	Explanation	Codes
Infection	Certain infections and parasitic diseases	A00-B99
Neoplasm	Neoplasm	C00-D48
Metabolic disease	Endocrine, nutritional and metabolic diseases	E00-E90
Mental disease	Mental and behavioral disorders	F00-F99
Neurologic disease	Diseases of the nervous system	G00-G99
Circulatory disease	Diseases of the circulatory system	I00-I99
Respiratory disease	Diseases of the respiratory system	J00-J99
Digestive disease	Diseases of the digestive system	K00-K93
Genitourinary disease	Diseases of the genitourinary system	N00-N99
Trauma	Injury, poisoning and certain other consequences of external causes	S00-T98
Others	Diseases of the blood and blood-forming organs and certain disorders involving the immune mechanism<br>Diseases of the skin and subcutaneous tissue<br>Diseases of the musculoskeletal system and connective tissue	D50-D89<br>L00-L99<br>M00-M99
NEC	Symptoms, signs and abnormal clinical and laboratory findings, NEC	R00-R99

NEC: Not elsewhere classified
